# Oblique Split Rib Graft Surgery in Primary and Secondary Septorhinoplasty

**DOI:** 10.29252/wjps.8.2.237

**Published:** 2019-05

**Authors:** Shahriar Loghmani, Alireza Loghmani, Fatemeh Maraki

**Affiliations:** 1 Department of Plastic Surgery, Ordibehesht Surgical Center, Isfahan University of Medical Sciences, Isfahan, Iran;; 2 Department of Dentistry, Ordibehesht Surgical Center, Isfahan University of Medical Sciences, Isfahan, Iran;; 3 Department of Operating Rroom, Isfahan university of Medical Sciences, Isfahan, Iran

**Keywords:** Oblique split method, Rib, Graft, Septorhinoplasty, Cartilage

## Abstract

**BACKGROUND:**

Rib cartilage is an outstanding material in reconstructive septorhinoplasty, especially in revision surgery with a low rate of complications compared to other materials. In this study, the results of oblique splitting of rib grafts were evaluated in 25 patients operated for primary and secondary septorhinoplasty.

**METHODS:**

The prospective case series were undertaken on 25 patients of saddle or crooked nose that referred to the senior author’s private office between January 2015 and November 2017. They had primary and secondary septorhinoplasty using autologous costal cartilage carved by the oblique split method (OSM). The postoperative follow-up period ranged from 3 to 36 months (Mean follow up of 19 months).

**RESULTS:**

The problems seen in patients were saddle-nose deformity in 16 cases, crooked-nose deformity in 3, crooked nose and saddle nose in 3 and implant infection, inverted V-pinch, destruction of septum in 3 more cases. After oblique split rib grafts surgery and 3-36 month follow-up (an average of 19 months), the operative outcomes were successful and no severe resorption, infection, warping or displacement were observed related to graft and patients were also satisfied, and there was no complication of the donor-site. The patients did not have any post-operative complications and no complain of nasal distortion during follow up period.

**CONCLUSION:**

OSM allowed obtaining large quantities of graft material without the risk of warping due to inclusion of both peripheral and central portions of the rib cartilage.

## INTRODUCTION

Nasal osteocartilagineous framework reconstruction is the foundation of augmentation rhinoplasty.^1^ For this purpose, different materials may be used such as autograft and allograft cartilages,^2^ that the former is preferred for many reasons such as less immune reaction and infection. It can be harvested from nasal septum, auricular concha or rib cartilages.^1^ For all types of aesthetic and functional requirements of rhinoplasty an abundant supply of cartilage is offered by the rib that provides reliable structural support. Unfortunately, the costal cartilage graft is a potent area to warp, which may cause nasal form distortion after surgery.^3^^,^^4^

Centrally, carved grafts have been shown with a decreased rate of warping compared with peripherally carved grafts; but with the present methods, the risk of warping could not be totally eliminated.^5^ A novel costal cartilage carving technique called “oblique split method (OSM),” introduced by Tastan *et al.* in 2013 to remove warping by dividing the graft obliquely could provide equal circumferential forces of contracture.^5^ The OSM provides a complete rib segment with an intact surface layer that can be easily carved. The flat surface of OSM graft is the cross section of the rib in which the diametrically opposed forces at all points are equal and may be said to be balanced.^6^^,^^7^

The elliptical OSM grafts preserve their straight configuration even after modifying into rectangular grafts or carved asymmetrically and/or the graft edges are beveled due to the balance in forces. This method can achieve straight grafts of varying thickness without any chance of warping.^6^^,^^7^ Therefore the results of this technique (OSM) were evaluated by the presentation of surgical technique and pre-, intra- and post-operative photographs of 25 patients operated for primary and secondary septorhinoplasty.

## MATERIALS AND METHODS

In this prospective case series, we analyzed data from 25 patients of saddle or crooked nose that referred to the senior author’s private office between January 2015 and November 2017 undergoing primary and secondary septorhinoplasty using autologous costal cartilage carved by the oblique split method (OSM). The postoperative follow-up period ranged from 3 to 36 months (Mean follow up of 19 months). All patients gave informed consent to participate in the study. A total of 25 patients (9 males and 13 females) were older than 18 years (mean age: 27.41±8.34 years).

**Fig. 1:**
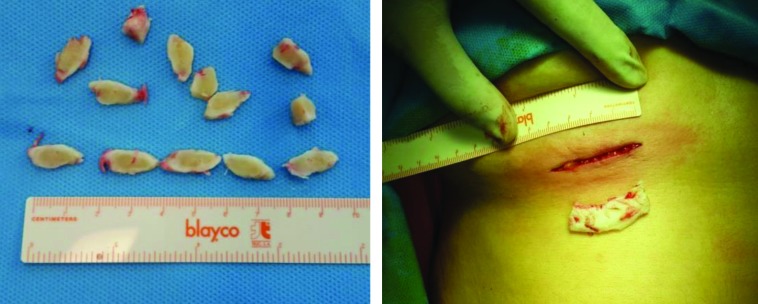
Costal cartilage graft harvested through a small incision (4 cm)

All septorhinoplasties were performed under general anesthesia using an open approach. Fifteen patients were primary cases including 7 with post-traumatic deformities and 8 presented as ethnic phenotype or unknown cause. There were 10 cases of secondary rhinoplasty that 6 ones had saddle nose due to aggressive and over-resection septorhinoplasty. The procedure included OSM technique and complications such as warping or displacement, resorption and donor site morbidity were evaluated in follow up duration by visiting the patients and clinical evaluation. the cartilage of fifth or sixth rib graft was harvested and for cosmetic reason, the incision line would be on infra-mammary line in woman and the length of skin incision should be 3 to 4 cm (Figure 1).^4^

The anterior perichondrium was harvested with rib cartilage and the costal cartilage was removed to be sculpted. Sometimes it is required for dorsal onlay graft that two pieces of graft were sutured end to end or by overlapping after the edges were adjusted.^5^ To camouflage the transition between the segments and for soft-tissue padding, costal perichondrium or rectus fascia could be sutured over the dorsal onlay graft. Splitting the cartilage was done with a surgical blade or dermatome blade in an oblique fashion to the long axis of the rib. A graft length roughly two times the superoinferior diameter would be obtained by dividing the cartilage at 45 degrees to the long axis and longer grafts may be achieved by dividing the rib at lower angles.^7^^,^^8^

The carved grafts were immersed for 30 minutes in isotonic sodium chloride solution and kept at room temperature. The resulting segments of cartilage might have trimmed edges or to be cut to the desired length and shape without causing the eventual warping. Through an open approach, septal mucosal flaps were elevated in a standard fashion and the cartilage remnants were exposed. Using a segmental reconstruction graft, the remained framework might be reinforced or rebuilt depending on the presence of a caudal or dorsal cartilage strip. Fine adjustment of the height and length of the new septum would be possible by segmental reconstruction. 

**Fig. 2:**
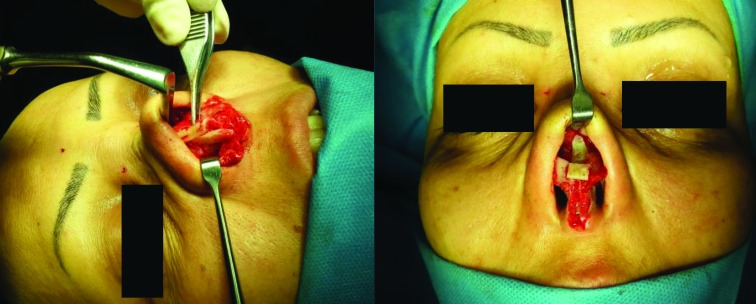
Two views during implantation of the rib cartilage graft

The grafts were sutured to each other and fixed to medial crura of lower lateral cartilages, caudal end of septum, and periosteum of anterior nasal spine, piriform aperture, and maxilla. All patients received antibiotics for 5 days starting the night before the operation, and washing surgical fields with gentamycin-saline solution continue throughout the surgery. Chest wall and nasal wounds were closed in layers, internal (doyle) and external nasal splint were removed 7 days after operation and the most donor site incisions were drained using a 6 mm suction drain for one day. Clinical evaluation was performed by patient observation and taking photos. Two views during implantation of the rib cartilage graft were shown in Figure 2.

## RESULTS

In the present study, 25 patients underwent primary and secondary septorhinoplasty; 15 cases were female and 10 cases were male with the mean age of 27.41±8.34 years (range: 18-49 years). The problem of these patients were saddle-nose deformity in 16 cases, crooked-nose deformity in 3, crooked nose and saddle nose in 3 and implant infection, inverted V-pinch, destruction of septum in 3 more cases. These problems have been resulted from many reasons such as trauma, previous septorhinoplasty, nasal fracture in childhood, ethnic, warping after rib graft and infection of alloplastic implant. 

**Fig. 3:**
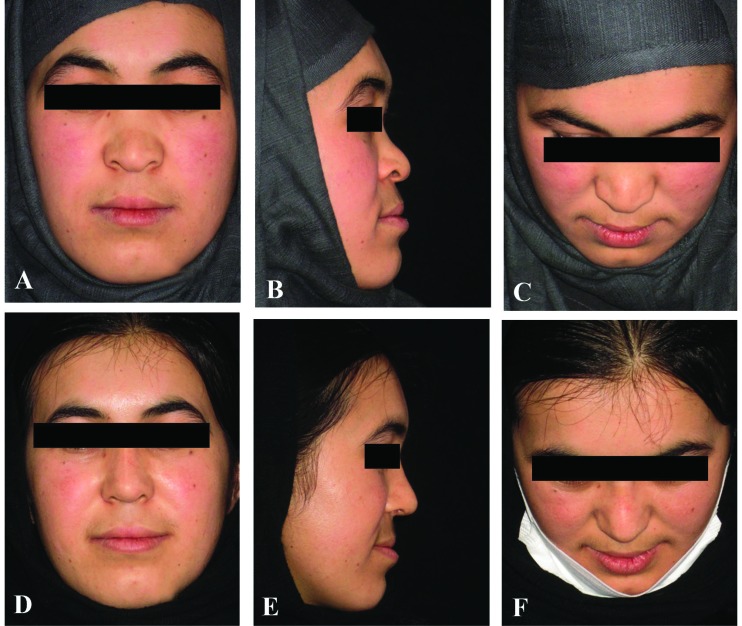
A 19-year-old woman with ethnic saddle nose deformity. The nasal dorsum was reconstructed with three layers of oblique splint rib and rectus fascia, using the 6^th^ costal cartilage, (A-C) Preopera­tive views. (D-F) Postoperative views at 6 months

**Fig. 4:**
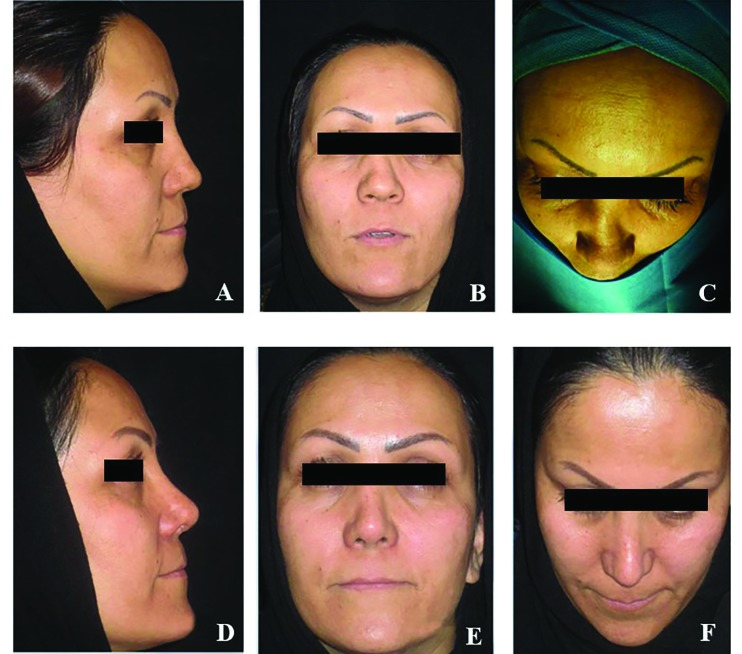
A 34-year-old woman with crooked nose deformity due to nasal fracture in childhood. The nasal septum was reconstructed, using the 6^th^ costal cartilage. (A-C) Preopera­tive views. (D-F) Postoperative views at 12 months

**Fig. 5:**
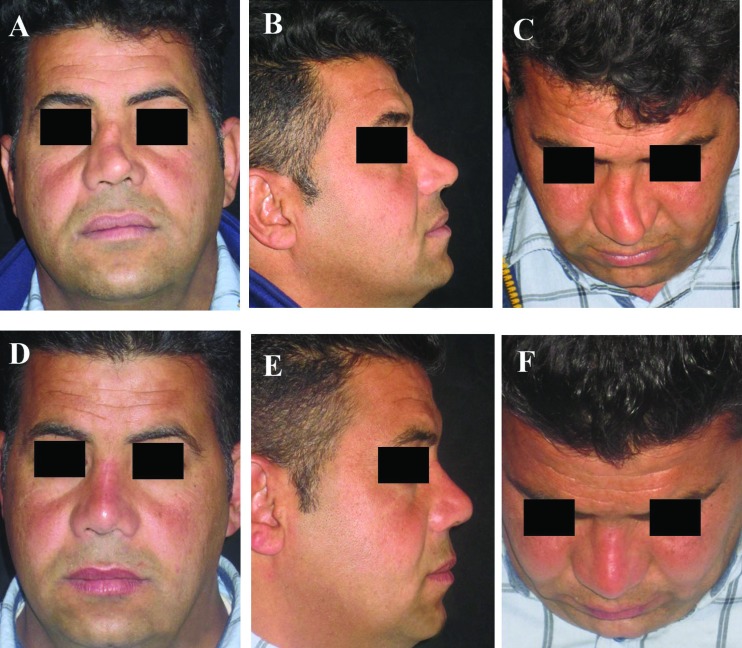
A 32-year-old man with warping after rhinoplasty for saddle deformity. The nasal framework was reconstructed using oblique splitting of the previous warped rib graft. (A-C) Preopera­tive views. (D-F) Postoperative views at 18 months

**Fig. 6:**
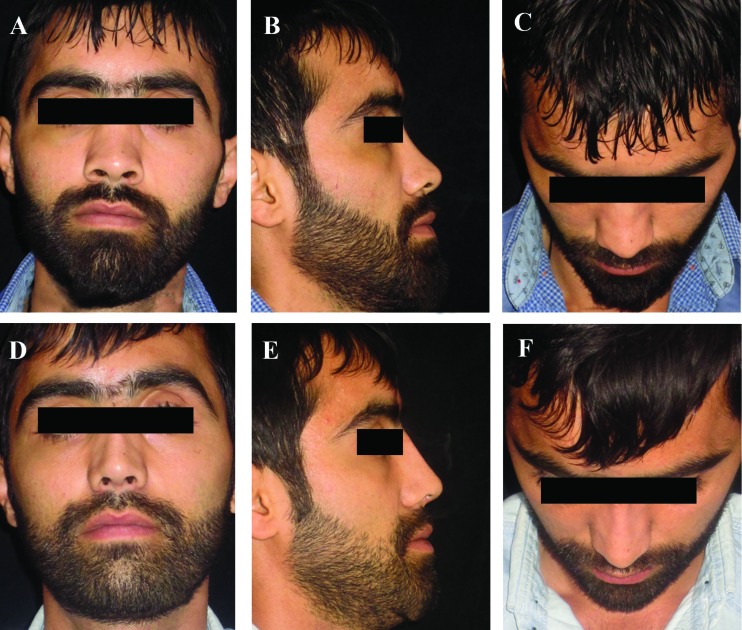
A 27-year-old man with saddle nose deformity due to panfacial fracture undergone primary rhinoplasty. The nasal dersum and septum was reconstructed with an oblique split rib graft, using the 6^th^ costal cartilage, and rectus fascia. (A-C) Preopera­tive views. (D-F) Postoperative views at 6 months

**Fig. 7:**
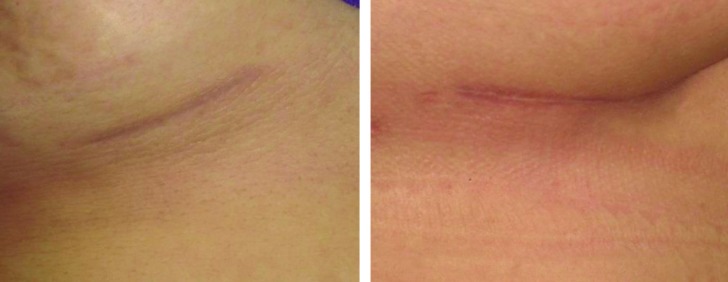
Donor-site scars one year after surgery

Ultimately, after oblique split rib grafts surgery and 3-36 month follow-up (an average of 19 months) after surgery, the operative outcomes of all patients were successful and no severe resorption, infection or warping or displacement were observed in relation to the transplant and patients were also completely satisfied (Figure 3-6). In the objective evaluation of the donor-site morbidity, all the cases showed good quality scars with no complications. The patients did not have any complications after surgery and no patient complained nasal distortion after operation during follow up period. The donor-site scars one year after surgery were demonstrated in Figure 7.

## DISCUSSION

Autologous costal cartilage is a commonly employed graft for septorhinoplasty. It is particularly favored as an abundant source of cartilage in cases of revision rhinoplasty in which more local sources (septum and concha) are depleted.^9^ Gibson and Davis emphasized the importance of reducing of warping by balanced costal cartilage cross sections, achieved by concentric carving as demonstrated in a retrospective study on 46 patients.^10^ Warping is related to distortion of graft material, which was shown later with structural deformity. Many different techniques have been utilized to minimize warping.^11^ The most frequently applied techniques were to delay grafting or immersing the graft in solution before shaping or insertion. 

The classic harvest technique can be modified to obtain central segment harvest.^7^ It is difficult to harvest a straight rod of cartilage from a curved rib segment without breaking the principles of balanced cross section. It is also technically not possible even if the rib segment is straight, because once the outermost layer has been cut off, the surface layer cannot be longer differentiated from the deeper zones and there is no orientation.^12^ Lopez *et al.* concluded that lower warping occurs in centrally cut cartilage grafts compared to peripherally cut grafts. They also found that the centrally cut pieces with a larger cross-sectional area, have less potential to warp.^13^ Warping is the most commonly reported complication with an almost rate of 10%, but with the use of these techniques, it varied substantially from 15% to 26.1%.^9^

Gunter *et al.* reported that internal stabilization of the rib cartilage grafts with Kirschner wire decreased the incidence of warping. However, some complications such as extrusion of Kirschner wire, infection, pain, and numbness of the anterior palatal mucosa were observed.^14^ In a study by Wilson *et al.*, they reported no significant differences for the amount of warping among patients treated with oblique split and concentric carving methods of costal cartilage graft carving.^15^ As the results of the present study showed in 6 cases (27.3%), the problems leading to saddle nose deformity were due to primary rhinoplasty and in fact, some patients requested for secondary rhinoplasty to resolve some problems such as uncorrected or refractory breathlessness or dissatisfaction with appearance and nasal congestion, but the risk of nose deformity was greater for the second time, and in this case, they had to undergo oblique split rib grafts surgery.

Adams *et al.* have shown decreased warping rates of rib cartilage, when the grafts were carved from the central portion of the harvested cartilage compared to the peripheral portions. When the central portion of the cartilage was carved, the forces would be balanced and the warping probability decreased. However, providing perfectly balanced grafts was often challenging.^12^ Yilmaz *et al*. evaluated 38 saddle nose patients augmented dorsally with rib cartilage grafts. Questionnaires filled out by the patients showed that the nasal shape was rated as very good and good by 76% of the patients. The remaining 24% reported the nasal shape unsuccessful, probably due to warping complication.^16^

In another study, warping was rated in 12 of 37 (32.4%) patients with severe saddle nose deformity with the three-step nasal reconstruction with costal cartilage. They preferred an osseocartilaginous dorsal graft from the ninth rib without internal Kirschner wire stabilization and could also minimize the risk of graft warping, because the effective portion of warping susceptible cartilage was greatly reduced.^17^ Ozturan *et al. *compared a standard grafting technique with the accordion technique (scoring the cartilage prior to insertion) in 23 patients with no reported cases of warping in the later technique. In this small series, no carving was performed on harvested grafts, instead of the grafts placed with the convex edge and oriented superiorly to utilize the natural curvature for shaping the nose (no cases of warping were reported).^18^

Moshaver *et al.* combined the use of central segment cartilage with Kirschner wires, but an 8.1% warping rate was seen in addition to Kirschner wire extrusion in 3 patients. It is thought that utilizing the central segment of costal cartilage minimizes warping, that’s why this method was employed in many studies. The majority of studies utilizing this technique reported less than 10% of warping rates; two reporting rates of greater than 10%, suggesting the effect of technique on reducing warping rates.^19^

Diced cartilage in fascia (DC-F) has revolutionized dorsal grafting. The basic concept is to dice cartilage into small bite (<0.5 mm), which can be put into a facial sleeve that is slipped into the dorsal defect. Danial *et al.* analyzed their experience gained from more than 400 DC-F grafts.^17^ Autogenous cartilage is the most favorable material for nasal augmentation with less functional stresses and demands for blood supply as well as it can be survived by delivery of nutrients by diffusion alone, while the chances of resorption are less.^20^ Finally, according to previous studies and the current study, oblique split rib grafts surgery is one of the most successful techniques of rib graft in rhinoplasty.

One of the limitations of our study was the lack of comparing the results of oblique split rib grafts operation with previous methods, because this comparison can show the preference better, and the higher success rate of this method in comparison to other methods. Therefore, it is suggested to compare the success rate of rhinoplasty in different surgical methods in future studies. Providing thin and straight grafts in septal cartilage–depleted patients is still challenge for the surgeon. The OSM is unique because it provides straight grafts as thin as septal cartilage. Also, with the OSM, it is possible to obtain large quantities of graft material without the risk of warping because the graft includes both peripheral and central portions of the rib. 

## CONFLICT OF INTEREST

The authors declare no conflict of interest.
